# Study protocol: Determining what young people with rheumatic disease consider important to research (the Young People’s Opinions Underpinning Rheumatology Research - YOURR project)

**DOI:** 10.1186/s40900-016-0037-8

**Published:** 2016-06-11

**Authors:** Suzanne Parsons, Kate Dack, Bella Starling, Wendy Thomson, Janet E. McDonagh, on behalf of the Barbara Ansell National Network for Adolescent Rheumatology (BANNAR)

**Affiliations:** 1 Public Programmes Team, Central Manchester University Hospitals NHS Foundation Trust and University of Manchester, Manchester Academic Health Science Centre, Manchester, UK; 2 Centre for Musculoskeletal Research and NIHR Manchester Musculoskeletal Biomedical Research Unit, University of Manchester, Manchester, UK; 3 Central Manchester University Hospitals NHS Foundation Trust, Manchester Academic Health Science Centre, Manchester, UK

**Keywords:** Adolescence, Participation, Protocol, Patient involvement

## Abstract

**Plain English summary:**

Involving young people in research about their health is increasingly recognized as being important to make sure that research is focused more on the needs of young people. However, at present, ideas about what should be researched and found out mainly come from researchers and health professionals like doctors and nurses rather than young people. Therefore, in the past, young people’s ideas about what should be researched in terms of rheumatic problems have not been explored. In this study, we will talk with groups of young people with rheumatic problems across the UK to explore what they think research into their health should focus on. We will also discuss with young people, if and how, they would like to be involved in shaping research into rheumatic problems. The findings from this work will help make sure that the views of young people with rheumatic problems influence the work of a group of researchers and health professionals who concentrate on rheumatology research. This group is called the Barbara Ansell National Network for Adolescent Rheumatology (BANNAR). A national young person’s advisory group will be set up to make sure that the beliefs and ideas of young people with rheumatic disease inform the work of the BANNAR.

**Abstract:**

**Background**

The involvement of people of all ages (including young people) in health-related research is now widely advocated but research priorities are still largely driven by professional agendas, with evidence from the adult literature reporting a mismatch between researcher and patient generated lists of research topics. To date, there have been no studies exploring the research priorities of young people with long term conditions including rheumatic disease. In this study, we will explore young people’s beliefs about their research priorities for rheumatic conditions and whether and how young people would like to become involved in the research process.

**Methods/Design**

We will hold up to 16 focus group discussions with young people (11–24 years) across England, Northern Ireland, Scotland and Wales. Two age groups will be recruited to the study, 11–15 year olds to represent early and mid-adolescence and 16–24 year olds to reflect late adolescence and emerging adulthood. Focus groups will be as interactive and engaging as possible, using a mixture of statement sorting and a research prioritization exercise to stimulate the discussion. Young people will be recruited via members of the Barbara Ansell National Network for Adolescent Rheumatology (BANNAR) and relevant national charities. Focus groups will be audiotaped and transcribed for analysis.

**Discussion**

This project will help ensure full representation from young people with rheumatic diseases in the development of a research strategy for BANNAR and will ultimately inform a young person’s led involvement strategy to facilitate the future ethical and meaningful involvement of young people in BANNAR members’ future research programmes. In addition, a national young persons’ advisory group will be established, the constitution and format of which will be determined by the young people themselves.

## Background

The involvement of people of all ages (including young people) in research is now widely advocated [1–8]. In the UK, the James Lind Alliance has reported that prioritization of research topics has been largely driven by professional agendas [9, 10] and has since led the way in involving patients in research priority setting, with the development of priority setting partnerships or PSPs. Priority setting partnerships bring together patients, carers and clinicians to identify and prioritize the “unanswered questions” about the effects of treatments that they agree are the most important [11]. However, there are few studies focusing on patient and public involvement (PPI) in priority setting [12] and even fewer which involve young people [13, 14]. Therefore, not surprisingly, mismatches between research evidence and the public’s views have been reported [15, 16]. As a consequence of these mismatches, the limited research funding available may be directed to research which people of all ages (including young people) do not value as highly as researchers. Musculoskeletal conditions are common reasons for young people consulting primary care [17, 18] and yet they are rarely involved in priority setting for research in this area of medicine.

Involving young people in research is an important ethical imperative [4, 19, 20, 21] and has been called for by young people themselves [1]. This has been reflected in changes in the grant and ethics application processes in the UK and is advocated by professional bodies [1–8]. The distinction between research participation and research involvement is often not clearly defined [22]. INVOLVE, the National Advisory Group supporting Public and Patient Involvement (PPI) in the NHS and Social Care [6] defines involvement as “where members of the public are actively involved in research projects and in research organisations”. Examples of public involvement are detailed in Table 1.

**Table 1:** Examples of Public and Patient Involvement in research

1. Involvement in identifying research priorities	
2. Co-design of interventions	
3. As members of a project advisory or steering group	
4. Commenting and developing patient information leaflets or other research materials	
5. Undertaking interviews with research participants	
6. User and/or carer researchers carrying out the research.	
7. Dissemination events of results	

Involving young people in identifying and prioritising research topics makes practice and policy more relevant to their needs leading to greater patient satisfaction, improvement in treatment adherence and/or better translation of research findings [20, 22–24]. In addition to priority setting, a key task in research is defining the research question and it is essential that those questions are relevant and meaningful to the lives of young people.

As well as being involved in defining research questions, young people can also work with researchers to develop and refine them further, decide on the most appropriate methodology to employ, the choice of research setting, the design of interventions, and the development and production of informational resources, consent and assent forms etc. Examples of existing practice in this area identified in a preparatory mapping exercise for the proposed study are detailed in Table 2.

**Table 2:** Models of Good Practice in the UK

Name of initiative	Website
Children’s Specialty theme in the National Institute for Health Research (NIHR) Clinical Research network [45, 46] *This initiative is supported by a national young person’s advisory group called GenerationR (R for Research) which is made up of several regional groups across the country which aim to support the design and delivery of paediatric research in the UK.*	http://www.crn.nihr.ac.uk/children/ http://generationr.org.uk
Centre for the Development and Evaluation of Complex Interventions for Public Health Improvement [47] *DECIPHer is a UKCRC Public Health Research Centre of Excellence. It brings together leading experts from a range of disciplines to tackle public health issues, with a particular focus on developing and evaluating multi-level interventions that will have an impact on the health and wellbeing of children and young people. Public involvement is undertaken in the Centre through employing a full time ‘Involving Young People Research Officer’ who supports and organises two groups: a young people’s advisory group (ALPHA: Advice Leading to Public Health Advancement) and a Public Involvement Steering Group made up of academics and practitioners with a sound understanding of public involvement.*	http://decipher.uk.net/
Royal College of Paediatrics and Child Health [48] & *Us is the RCPCH’s platform for children, young people, parents, carers and families to join the RCPCH in improving healthcare for young patients. The aim is to provide a variety of and flexible ways for, people to share their views and experiences, which will ensure the patient voice is at the heart of our work. An Infant, Children and Young Persons Research Charter is currently being developed as part of this work.*	www.rcpch.ac.uk
Association for Young People’s Health [49] *AYPH is a progressive charity and membership forum, creating a focus for everyone working in the field of young people's health across the UK. They are currently working to develop a youth-led research agenda for health.*	www.ayph.org.uk
University of Central Lancashire - Centre for Children and Young People’s Participation [50] *Based at The University of Central Lancashire’s School of Social Work, and founded in 2008 by Professors Nigel Thomas and Andy Bilson, the Centre for Children and Young People’s Participation is the only research centre devoted to this theme, and has an international reputation for research and knowledge exchange. The Centre also specialises in supporting children and young people to propose, plan and carry out their own research, as well as being partners on adult research projects.*	http://www.uclan.ac.uk/research/explore/groups/centre_young_people_participation.php
Transition - The United Progression (UP) - Young people’s Involvement Group [51] *UP is a working group of young people with health needs, or personal experience of young people’s health needs, who work with the Project Management Board of the 5 year NIHR Transition Research Programme examining how health services can contribute most effectively to facilitating successful transition of young people with complex health needs from childhood to adulthood. UP was established to advise the full programme in addition to developing for their own youth led work stream*.	http://research.ncl.ac.uk/transition/
Participation Works [52] *Participation Works is a consortium of seven national children and young people's agencies that enables organisations to effectively involve children and young people in the development, delivery and evaluation of services that affect their lives.*	http://www.participationworks.org.uk/
National Youth Agency [53] *The National Youth Agency’s Young Researcher Network (YRN) has launched toolkits to help young people undertake youth-led research and promote their findings*	http://www.nya.org.uk/resource_category/young-researchers-network/
Youth Health Talk (involvement in clinical trials) [54] *This website enables patients to share their experiences online. ‘Youthhealthtalk.org’ and ‘Healthtalkonline.org’ come from a unique partnership between The DIPEx charity and Oxford University’s Department of Primary Care.*	http://healthtalkonline.org/young-peoples-experiences
National Children’s Bureau [55] *The NCB involve young people in their research because they believe it improves the quality of the research and makes it more relevant and more persuasive for policymakers and practitioners. This includes training of young people for such involvement.*	http://www.ncb.org.uk/
Council for Disabled Children [56] *The VIPER project, consists of 16 young disabled people, aged 12 to 22, from across England. Resources developed as part of this project are available on the website*	http://viper.councilfordisabledchildren.org.uk/home/
INVOLVE [6] *INVOLVE is funded by the National Institute for Health Research in the UK (NIHR) to support public involvement in NHS, public health and social care research.*	http://www.invo.org.uk/ http://www.invo.org.uk/find-out-more/involving-children-and-young-people/
Nuffield Council on Bioethics [54] *The Nuffield Council on Bioethics is independent body advising policy makers and stimulating debate in bioethics and have recently specifically addressed issues specific to children and young people*	http://nuffieldbioethics.org/project/children-research/

There are numerous benefits to involving young people in undertaking research, including: benefits to research and development (e.g., introduce young people’s perspectives), benefits to research dissemination (e.g., can help achieve bigger impact) and benefits to the young people who get involved (e.g., transferable skills, valuable experience and recognition) [25]. However, the evidence base to support the impact of PPI in health research irrespective of age although rapidly developing, remains limited and more research in this area is needed [26–29].

### Need for research in this area

Some researchers have reported challenges to collaborating with young people in health research [22, 30, 31] including workload, recruitment, ethics, aspects of power and impact on research quality. Gaining a greater understanding of how young people see their potential roles as active research partners in research into long term conditions, is needed to ensure that research is designed that focuses on the issues and outcomes of importance to young people.

The authors explored the existing involvement of young people in rheumatology research programmes by surveying members of the British Society of Paediatric and Adolescent Rheumatology (BSPAR) in 2013/2014. All BSPAR members (*N* = 247) were sent a 9 item online questionnaire embedded in the regular member’s newsletter. Thirty one members responded representing 25 rheumatology units, of which 15 had at least one full time consultant paediatric rheumatologist and 10 had a paediatrician with an interest in rheumatology (*n* = 5) and/or adult rheumatologist (*n* = 5). There was just one established youth advisory panel amongst those who responded (for 10–19 year old age group) and seven centres reported ad hoc groups. In five centres, patient involvement was specified in the job description of one member of their team. The majority of those who responded did not involve young people in their research. Further details of the areas where young people aren’t involved are described in Table 3. Involvement is likely to be even worse for young adults (16–24 years) with childhood onset rheumatic disease being cared for in the adult healthcare environment. A study across the whole of paediatric and adult rheumatology is required to better quantify both participation and involvement needs within this age group across the UK.

**Table 3:** Lack of involvement of young people in Rheumatology research – From BSPAR survey data

Nature of involvement	% not involved
Setting research agendas	80 %
Development of protocols	64 %
Design of interventions	60 %
Membership of advisory boards	64 %
Dissemination of results	60 %
As co-researchers	80 %
Recruitment of research staff who will have direct contact with young people	80 %

The Barbara Ansell National Network for Adolescent Rheumatology (BANNAR) was established in 2013 by Arthritis Research UK (http://bannar.org.uk). BANNAR aims to establish a network of research interested rheumatology professionals. It also aims to ensure that every young person in the UK has the best chance possible to benefit from developments in the field of adolescent and young adult rheumatology. The network is particularly interested in including the input of young people into developing its research priorities and the projects conducted by those professionals within BANNAR. It is important that the involvement of young people in the work of BANNAR is meaningful, ethical, well-structured and communicated. A key area of focus of this network is to address the challenges transitional care presents to both young people and families, the health service itself, as well as research programmes which span the adolescent- young adult age span [3, 32]. Particular research challenges include the recruitment and retention of young people at a time they are facing multiple transitions; the relationship between child and adult outcome measures, and the tracking of young people as they move into adult care and eventually away from the parental home.

We felt that it was important to publish this protocol, as although there is currently much involvement work being undertaken in healthcare and healthcare research [7] (although less specifically with young people), the work that is undertaken is often reported with minimal detail, meaning that an understanding of how to involve a range of patient populations in healthcare research has been slow to grow.

Publication of protocols, such as the one described here, are likely to play an important role in increasing the general understanding of the range of approaches available to involve young people and the rationale behind why to employ specific approaches.

### Aims and objectives of the YOURR project

This study aims to explore the beliefs of young people in the UK about their research priorities for rheumatic conditions and to determine whether and how young people would like to become involved in the research process.

The project has four objectives:

1. To explore the experience of research participation and research involvement of young people with rheumatic conditions
2. To identify the research priorities of young people with rheumatic conditions and explore how these can be used to refine and prioritise the future research strategy of the BANNAR
3. To explore young people’s beliefs about involvement in research, e.g., whether they feel they should be involved, what helps and hinders their involvement and what they believe are the best ways of involving them
4. To use the study findings to develop a young person’s involvement strategy and forum to facilitate the meaningful involvement of young people in the future research work of the BANNAR.

## Methods/Design

This is a qualitative study of young people with rheumatic disease, up to 16 focus groups will be conducted (Eight with 11–15 year olds and eight with 16–24 year olds). The age ranges were chosen to reflect adolescent developmental stages i.e., early and mid-adolescence (11–15 years) and late adolescence and young adulthood (16–24 years).

If data saturation is reached we may not conduct all 16 focus groups. However, as far as possible we will conduct focus groups in all four nations of the UK. This is because service organization is likely to vary across England and the devolved nations, and we have hypothesized that this may impact on the research priorities as well as the research experience of young people.

Up to eight young people will take part in each focus group. The optimum size for a focus group is generally considered six to eight participants. However, larger groups with this age range could potentially be frustrating for participants due to insufficient opportunities to speak, particularly if they are initially shy and/hesitant. We will therefore aim to recruit 8 young people to allow for 1–2 drop outs on the day [33].

### Participants

Young people with a range of chronic conditions will be recruited including inflammatory arthritides (juvenile idiopathic arthritis, inflammatory bowel disease associated arthritis, adult rheumatoid arthritis, ankylosing spondylitis, psoriatic) in addition to connective tissue diseases (such as SLE scleroderma, vasculitides), chronic recurrent focal osteomyelitis, and chronic idiopathic pain syndromes. Although we had identified established groups of young people already involved in similar advisory groups in an earlier phase of the research (Table 2), the focus of the work was to establish a rheumatology-specific research agenda.

Adolescent and emerging adult [34] development is now understood to extend from the age of 10 and onset of puberty right through to the maturation of the prefrontal cortex in the mid-twenties [35, 36]. Young people in this 10–24 year old age range therefore represent a wide range of perspectives and biopsychosocial abilities all of which are important to consider when involving young people in research. Young people in the upper age range are not a representative voice for young people in the early adolescent developmental stage, the latter whom are least often represented in current practices. This is why we have included the relatively wide age range of 11–24 year olds in this study. Given the limited resources available for this project we were only able to split the sample using one key sampling variable (either age or gender). We chose age as we felt that having young people potentially aged 11 to 24 years in one focus group would inhibit the group discussion, impacting negatively on the group dynamics and young people’s ability and confidence to participate.

### Recruitment

The different age groups will require different recruitment approaches. This is because 16–24 year olds can independently consent to take part in the study, but consent from parents for 11–15 year olds to be approached about the study is required. Once parental consent is obtained, 11–15 year olds assent to take part will then be obtained.

Participants will be recruited either in hospital clinics, or via advertisements on medical research charity websites.

For clinic recruitment, − hospital consultants and/or senior rheumatology team members will be provided with the study inclusion and exclusion criteria and will be asked to provide a study information sheet containing details of the study aims and processes, and contact details of the research team to a broad range of potential participants who vary in terms of their age, gender, ethnicity, condition experienced, prior research experience (including research naïve young people) and socio-economic status.

Young people will also be recruited via medical research charities to ensure that the study involves those who are under the care of rheumatologists unrelated to BANNAR.

Parents of those aged 11–15 years old will be asked to reply to the advertisement and those aged 16 years and older will be able to reply to the advertisement in their own right.

### Focus groups

Up to eight young people will take part in each focus group.

After potential participants and their parents (where appropriate) have expressed interest in the study they will receive a telephone call from the research team to arrange a time for their participation and to answer any further questions they may have. If their parents agree, young people aged 11–15 will be asked to sign an assent form indicating their assent to participate [37]. Consent and assent forms will be signed at the beginning of each focus group. However, participants will be told by the facilitator that they are free to leave at any time if they wish, and that if they do that their previous comments will not be included within the study dataset.

Parents of those aged 11–15 will be able to accompany participants but will wait in a separate room within the focus group venue to allow young people to speak as freely as possible.

Focus groups will be moderated by SP and/or JMcD who have no direct involvement in the clinical care of any participant.

#### Focus group interview organisation

Focus groups will be held at easily accessible locations (i.e., centrally located with high levels of building accessibility, e.g., lifts and good signage) and all expenses will be covered for participants and their parents if they accompany their children. Refreshments will also be provided and a certificate to acknowledge contribution. All focus group participants will also receive a £20 gift voucher to compensate them for their time. Focus groups will be organized at times (times of year and times of the week) that are convenient to young people, to ensure that as many young people feel able to and can participate as possible.

#### Focus group sampling

We will identify maximum variety purposive samples of young people in terms of age, gender, ethnicity, rheumatic condition and socio-economic status. Up to ten groups will be conducted across England, two in Northern Ireland, two in Scotland and two in Wales (Table 4).

**Table 4:** Distribution of focus groups across the UK

England	Northern Ireland	Scotland	Wales
5 groups of 11–15 year olds	1-group of 11–15 year olds	1 group of 11–15 year olds	1 group of 11–15 year olds
5 groups of 16–24 year olds	1 group of 16–24 year olds	1 group of 16–24 year olds	1 group of 16–24 year olds

In terms of recruitment we will take the following steps to ensure that we can engage as diverse a group of young people as possible:

1. Research will be undertaken in all four nations of the UK to ensure that variation is captured in terms of access to healthcare and the impact that this might have on young people’s research priorities
2. Young people will be recruited from clinical departments which have a strong research and/or involvement culture and those that do not as was indicated by the BSPAR survey undertaken by the research team as part of the preparatory work for this study.

#### Focus group topic guide

Focus groups will be designed to be as interactive as possible; using a variety of visual stimuli and exercises to maintain participants’ interest.

Focus groups will last for up to 90 min and will be organised as follows:

- **Introductions (5 min)** – includes a brief overview of the project and an outline of what will happen in the discussion group.
- **Ice-breakers (10 min) -** ‘Getting to know you’ activity in pairs.
- **Beliefs about the research process and about getting involved in research (15 min)**– The group will be given a series of statements about the research process, and about getting involved in research and will be asked to sort these into those that they agree with and those that they disagree with. They will also be asked to discuss their experiences and beliefs about taking part in research.
- **Research priorities for young people with rheumatic conditions (45 mines)**
- The Research team will briefly describe to the group the areas which are currently researched in rheumatology conditions. These are:
  - ***1. Basic Science*** **;** ***2. Clinical Medicine and Science*** **;** ***3. Psychosocial; 4. Health services;*** **5. Public Health**
- Within each area, participants will be asked to give their ideas regarding what it is important for researchers to research. They will be also asked whether there are other areas researchers should focus on.
- The group will then be asked to order the research areas from those which they believe should receive the most research funding to those that they believe should receive the least, and to discuss the rationale for their choices.
  - **◦ Getting involved in research (10 min) –** Finally, the group will be asked to discuss their ideas about the involvement forum and how they should be involved.

Close of focus group

#### Data management

Data will be analysed using the FRAMEWORK approach to qualitative data analysis [38]. We have decided to use this approach because of its transparent nature and utility in a research team. It also has strength in facilitating both between and within case analysis. The data management and analysis process will be facilitated by the use of NVIVO qualitative software [39].

### Ethics

The study received ethics approval from Newcastle and North Tyneside NRES Committee. Ethical committee approval is not always considered necessary for collaborative patient and public involvement work in the UK. However, we decided to apply for approval, due to the age of our participants and also as the majority of participants will be identified via clinical units at UK Hospitals. Therefore, applying for ethical and research governance approval for the study provided an objective assessment of our study procedures, and will also help to facilitate our communication of the study across clinical units involved in recruitment.

## Discussion

As described earlier, there is currently much involvement work being undertaken in healthcare and healthcare research [7] (although less specifically with young people). However, the work that is undertaken is often reported with minimal detail, meaning that an understanding of how to involve a range of patient populations in healthcare research has been slow to grow. Also as there is currently not as much involvement work undertaken with young adults (16–24 years) we felt that this further strengthened the argument for this protocol paper. Without such protocols it may be difficult to further increase understanding of patient and public involvement and researcher’s willingness to involve patients and the public in their work. Therefore, we believe that it is important to understand the considerations required when exploring the views of specific population groups such as young people, i.e., how and when best to involve groups.

Therefore, we felt that it would be useful to publish the protocol for this priority setting study with young people to describe and discuss our approach to this work, in the hope that it may be useful to people considering undertaking similar work in the future.

In the UK, there is increasing advocacy for the involvement of patients and members of the public in health related research and service development [7]. However, evidence of the involvement of young people in the initial stages of research, namely setting research agendas and prioritisation of research topics, is limited [12–14]. This finding is further supported by the results of our survey of rheumatology professionals. Of note however, there was a poor response rate although all the major UK paediatric rheumatology units were represented. Due to the nature of the topic, it was likely that professionals were more likely to reply if they were aware of such activities and/or interested in the area and conversely not respond if they were unaware or not specifically interested. Clinton-McHarg et al. reported a value-weighting approach to determining research priorities for young people with haematological cancer which involved both consumers and professionals. However, only 10 of the 20 consumers were young people in the emerging adulthood developmental stage, i.e., not adolescent per se and their priorities were not considered separately from other consumers [13]. In another research priority setting exercise to improve the health of children and young people with neurodisability, consumers were involved but the majority of these were parents with only four young people involved throughout the process [14]. To the authors’ knowledge, this current study is the first to consider the research priorities specifically of young people including adolescents, with long term conditions.

Our original hypothesis is that by involving young people from the outset, we will develop a research agenda resonant with their lives and thus improve the outcomes of future BANNAR research programmes by improving recruitment and retention. For example, Nguyen B et al. et al. reported 88 % retention at 2 years in an obesity project following involvement of overweight and obese adolescents (13–16 year olds) in the design of the intervention [40]. Jamal et al. reported the positive impact of consulting with young people to inform systematic reviews including the assurance that issues important to young people were considered and that “early signals” of these issues were flagged for the synthesis [41]. By potentially enhancing participation, we may also see improved health outcomes. Mattila et al. reported young people who are non-respondents to research participation have poorer health outcomes overall as adults [42]. Likewise it will be important to identify and measure experiences and outcomes that are important to young people and not just to their caregiver and/or health professionals caring for them.

### Discussions of methods

#### Use of existing groups

As the aim of this work is to inform the development of the research programme of a network of researchers and clinicians focusing on rheumatic conditions, we explored the views of young people with these conditions. Our initial audit revealed one established group as well as several ad hoc youth advisory groups in the UK Rheumatology community. In acknowledgement of the importance of learning from existing groups of young people with and without other long term health conditions which have been established for similar purposes, we conducted a mapping exercise as a preparatory element of this project where the project team talked with those who currently run YPAGs to explore their experiences and beliefs regarding what they felt was good practice when involving young people [43]. The information gathered has formed the basis of interim guidance to the BANNAR prior to the results of this study being available and a resource document which will be updated annually [44].

It should be recognised that the YOURR project is a research study in itself so young people may decline involvement. In order to at least partially address this, we aim to recruit young people who currently have little experience of research participation or who have participated but don’t want to in the future to understand their views. This may help us to gain insight into young people’s potential reluctance to participate in future research.

We have chosen to use focus groups as the main approach to data collection within this study. We felt that this was the most appropriate approach as this is an area about which currently little is known. Therefore exploring the breadth of issues within a group setting seemed to be the most appropriate approach to take. We also felt that using focus groups with this age group would ensure that there was not too much focus on particular individuals’ views which some may find intimidating. Specific activities, e.g., the sorting exercise to discuss research beliefs will be used to facilitate this further, particularly with respect to involving those who feel less comfortable with group participation.

However, we also appreciate that there may be some disadvantages to using a focus group approach. For example, using focus groups may have meant that those young people who do not feel comfortable talking in a group may be less likely to participate. Also some young people may decide not to take part due to being uncomfortable with talking about their condition within a group, and not wanting to be identified as someone with a chronic rheumatic condition. However, we do state in the project information sheet that the discussions will not focus on individual experiences of rheumatic disease, Also using focus groups as the main approach to data collection may make it difficult for young people to participate if they are ill on the day of the group, i.e., it will be easier to rearrange a one to one interview rather than focus group participation. Rheumatic conditions for many young people can be unpredictable and therefore it is likely that some young people may agree to take part but may have to drop out on the day if they are unwell. Using a focus group approach may therefore make it difficult to rearrange participation for participants in this situation. However, data collection during acute illness episodes in always challenging irrespective of method.

In the proposed future involvement work following the completion of this study, it will be important to consider a range of ways in which young people can be involved (including the use of social media) to ensure that all of the potential challenges to participation detailed above are tackled. For example we will use a wider range of consultation methods in the future involvement work to ensure that the perspectives of those who are less comfortable in talking in a group are also considered. It is hoped that young people will lead this aspect of development work.

In conclusion, this project aims to involve young people with rheumatic diseases in the development of a national research strategy and will ultimately inform a young person’s led involvement strategy to facilitate the future ethical and meaningful involvement of young people in future research programmes of BANNAR. In addition, it will contribute to the currently limited evidence base of the impact and evaluation of the involvement of young people in health research

## Abbreviations

BANNAR, Barbara Ansell National Network for Adolescent Rheumatology; BSPAR, British Society for Paediatric and Adolescent Rheumatology; YOURR, Young People’s Opinions Underpinning Rheumatology Research

## References

[1] Bate J, Ranasinghe N, Ling R, Preston J, Nightingale R, Denegri S. Public and patient involvement in paediatric research. Arch Dis Child Educ Pract Ed. 2016. doi:10.1136/archdischild-2015-309500. [Epub ahead of print]. 10.1136/archdischild-2015-309500 26802108

[2] Department of Health (2008). Real involvement: working with people to improve services.

[3] McDonagh JE, Bateman B (2012). Nothing about us without us. Research involving young people. Arch Dis Child Educ Pract Ed.

[4] Royal College of Paediatrics and Child Health and Young Persons Health Special Interest Group (2010). Not just a phase. A guide to the participation of children and young people in health services.

[5] Modi N, Vohra J, Preston J, Elliott C, Van’t Hoff W, Coad J, Gibson F, Partridge L, Brierley J, Larcher V, Greenough A, for a Working Party of the Royal College of Paediatrics and Child Health (2014). Guidance on clinical research involving infants, children and young people: an update for researchers and research ethics committees. Arch Dis Child.

[6] National Institute of Health Research. INVOLVE: promoting public involvement in NHS, public health and social care research. 2010. http://www.invo.org.uk. Accessed 6 Mar 2016.

[7] National Institute for Health Research: Going the extra mile: improving the nation’s health and wellbeing through public involvement in research. March 2015 (http://www.nihr.ac.uk/documents/about-NIHR/NIHR-Publications/Extra%20Mile2.pdf) Accessed Apr 2016.

[8] Association of Medical Research Charities. Natural Ground. Paths to patient and public involvement for medical research charities. October 2009 (http://www.amrc.org.uk/sites/default/files/doc_lib/2009-10%20Natural%20ground.pdf) Accessed Apr 2016.

[9] Oliver S, Gray J (2006). A bibliography of research reports about patients’, clinicians’ and researchers’ priorities for new research.

[10] Stewart R, Oliver S (2008). A systematic map of studies of patients’ and clinicians’ research priorities.

[11] James Lind Alliance Guidebook, Version 6 – (http://www.jla.nihr.ac.uk/guidebook) Accessed Apr 2016.

[12] Gagnon M, Desmartis M, Lepage-Savary D (2011). Introducing patients’ and the public’s perspectives to a health technology assessment: a systematic review of international experiences. Int J Technol Assess Health Care.

[13] Clinton-McHarg T, Paul C, Sanson-Fisher R, D’Este C, Williamson A (2010). Determining research priorities for young people with haematological cancer: a value-weighting approach. Eur J Cancer.

[14] Morris C, Simkiss D, Busk M, Morris M, Allard A, Denness J, Janssens A, Stimson A, Coghill J, Robinson K, Fenton M, Cowan K (2015). Setting research priorities to improve the health of children and young people with neurodisability: a British Academy of Childhood Disability-James Lind Alliance Research Priority Setting Partnership. BMJ Open.

[15] Tallon D, Chard J, Dieppe P (2000). Relation between agendas of the research community and the research consumer. Lancet.

[16] Tong A, Sainsbury P, Carter SM, Hall B, Harris DC, Walker RG, Hawley CM, Chadban S, Craig JC (2008). Patients’ priorities for health research: focus group study of patients with chronic kidney disease. Nephrol Dial Transplant.

[17] Churchill D, Allen J, Pringle M, Hippisley-Cox J, Ebdon D, Macpherson M, Bradley S (2000). Consultation patterns and provision of contraception in general practice before teenage pregnancy: case-control study. BMJ.

[18] Haller DM, Sanci LA, Patton GC, Sawyer SM (2007). Toward youth friendly services: a survey of young people in primary care. J Gen Intern Med.

[19] UNICEF (2009). Summary of the United Nations Convention on the Rights of the Child.

[20] Nuffield Council on Bioethics (2015). Children and clinical research: ethical issues.

[21] Kirby P (2004). A guide to actively involving young people in research: for researchers, research commissioners, and managers’.

[22] Bird D, Culley L, Lakhanpaul M (2013). Why collaborate with children in health research: an analysis of the risks and benefits of collaboration with children. Arch Dis Child Ed Pract.

[23] Richards T (1999). Patients’ priorities. Br Med J.

[24] Entwistle VA, Renfrew MJ, Yearly S (1998). Lay perspectives: advantages for health research. Br Med J.

[25] Nuffield Council on Bioethics, London http://nuffieldbioethics.org/project/children-research/ (last Accessed 26 June 2015).

[26] Staniszewska S (2009). Patient and public involvement in health services and health research: a brief overview of evidence, policy and activity. J Res Nurs.

[27] Staniszewska S, Brett J, Mockford C, Barber R (2011). The GRIPP checklist: strengthening the quality of patient and public involvement reporting in research. Int J Technol Assess Health Care.

[28] Brett J, Staniszewska S, Mockford C, Herron-Marx S, Hughes J, Tysall C, Suleman R (2014). Mapping the impact of patient and public involvement on health and social care research: a systematic review. Health Expect.

[29] Brett J, Staniszewska S, Mockford C, Herron-Marx S, Hughes J, Tysall C, Suleman R (2014). A systematic review of the impact of patient and public involvement on service users, researchers and communities. Patient.

[30] van Staa A, Jedeloo S, Latour JM, Trappenburg MJ (2010). Exciting but exhausting: experiences with participatory research with chronically ill adolescents. Health Expect.

[31] Holland S, Renold E, Ross NJ, Hillman A (2010). Power, agency and participatory agendas: a critical exploration of young people’s engagement in participative qualitative research. Childhood.

[32] McDonagh JE, Kelly DA (2010). The challenges and opportunities for transitional care research. Pediatric Transplant.

[33] Steward DW, Shamdasani PM (1990). Focus groups: theory and practice.

[34] Arnett JJ (2000). Emerging adulthood: a theory of development from the late teens through the twenties. Am Psychol.

[35] Steinberg L (2010). A behavioral scientist looks at the science of adolescent brain development. Brain Cogn.

[36] Colver A, Longwell S (2013). New understanding of adolescent brain development: relevance to transitional healthcare for young people with long term conditions. Arch Dis Child.

[37] Health Research Authority. Health Research Authority Consent and participant information sheet preparation guidance. (http://www.hra-decisiontools.org.uk/consent/) Accessed Apr 2016.

[38] Ritchie J, Lewis J (2003). Qualitative research practice- a guide for social science students and researchers.

[39] NVIVO - http://www.qsrinternational.com/products_nvivo.aspx (Accessed 8 June).

[40] Nguyen B, Shrewsbury VA, O’Connor J, Steinbeck KS, Hill AJ, Shah S, Kohn MR, Torvaldsen S, Baur LA (2013). Two-year outcomes of an adjunctive telephone coaching and electronic contact intervention for adolescent weight-loss maintenance: the Loozit randomized controlled trial. Int J Obes (Lond).

[41] Jamal F, Langford R, Daniels P, Thomas J, Harden A, Bonell C. Consulting with young people to inform systematic reviews: an example from a review on the effects of schools on health. Health Expect. 2014. [Epub ahead of print]. 10.1111/hex.12312 PMC5810726 25470115

[42] Mattila VM, Parkkari J, Rimpela A (2007). Adolescent survey non-response and later risk of death. A prospective cohort study of 78609 persons with 11 year follow-up. BMC Public Health.

[43] Dack K, Williams H, Parsons S, Thomson W, McDonagh JE, on behalf of BANNAR. Summary of good practice when involving young people in health-related research. 2015. (http://bannar.org.uk) Accessed Apr 2016.

[44] McDonagh JE, Parsons S. BANNAR guidance for Involvement of Young People in Rheumatology Research. Interim Statement. 2015. (http://bannar.org.uk) Accessed Apr 2016.

[45] Children’s Specialty theme in the Clinical Research Network, NIHR, UK http://www.crn.nihr.ac.uk/children/ (last Accessed 26 June 2015).

[46] Generation R. Children’s Specialty theme in the Clinical Research Network, NIHR, UK http://generationr.org.uk (last Accessed 26 June 2015).

[47] Centre for the development and evaluation of complex interventions for public health improvement http://decipher.uk.net/ (last Accessed 26 June 2015).

[48] Royal College of Paediatrics and Child Health www.rcpch.ac.uk (last Accessed 26 June 2015).

[49] Association for Young People’s Health www.ayph.org.uk (last Accessed 26 June 2015).

[50] Centre for Children and Young People’s Participation, University of Central Lancashire http://www.uclan.ac.uk/research/explore/groups/centre_young_people_participation.php (last Accessed 26 June 2015).

[51] Transition - The United Progression (UP) - Young people’s Involvement Group, NIHR http://research.ncl.ac.uk/transition (last Accessed 26 June 2015).

[52] Participation Works http://www.participationworks.org.uk (last Accessed 26 June 2015).

[53] National Youth Agency, UK http://www.nya.org.uk/resource_category/young-researchers-network/ (last Accessed 26 June 2015).

[54] Youth Health Talk, University of Oxford http://healthtalkonline.org/young-peoples-experiences (last Accessed 26 June 2015).

[55] National Children’s Bureau http://www.ncb.org.uk/ (last Accessed 26 June 2015).

[56] VIPER Project, Council for Disabled children http://viper.councilfordisabledchildren.org.uk/home/ (last Accessed 26 June 2015).

